# Effectiveness and Safety of Levothyroxine Tablets Combined with Iodine-131 in the Treatment of Thyroid Cancer

**DOI:** 10.1155/2022/3676886

**Published:** 2022-06-02

**Authors:** Yang Bai, Jian Jin, Yonghong Liu, Buyong Zhang, Bo Zhang, Jie Li

**Affiliations:** Fourth Department of Thyroid Mammary, Cangzhou Central Hospital, 16 Xinhua West Road, Cangzhou, Hebei, China

## Abstract

**Objective:**

To explore the effectiveness of levothyroxine tablets combined with iodine-131 in thyroid cancer patients after radical thyroidectomy and the effect on their serum thyroglobulin (Tg) and thyroglobulin antibody (TgAb) levels.

**Methods:**

A total of 70 thyroid cancer patients receiving radical thyroidectomy in our hospital from July 2015 to April 2016 were recruited and were assigned via different treatment methods (1 : 1) to receive either levothyroxine tablets (observation group) or levothyroxine tablets plus iodine-131 (control group). Outcome measures included treatment efficiency, 1, 3, and 5-year recurrence and metastasis, serum Tg and TgAb levels, postoperative survival, and adverse reactions.

**Results:**

The total effective rate of treatment in the control group was significantly higher than that in the observation group (*P* < 0.05). There was no significant difference in cancer recurrence and metastasis rate between the two groups one year postoperatively (*P* > 0.05). The rate of cancer recurrence and metastasis in the control group was significantly lower than that in the observation group 3 and 5 years after surgery (*P* < 0.05). Before treatment, there was no significant difference in serum Tg and TgAb levels between the two groups (*P* > 0.05). After treatment, serum Tg and TgAb levels decreased in both groups, with lower results in the control group (*P* < 0.05). There was no significant difference in the 1 and 3-year survival rates between the two groups (*P* > 0.05). The 5-year survival rate in the control group was significantly higher than that in the observation group (*P* < 0.05). There was no significant difference in adverse reactions between the two groups (*P* > 0.05).

**Conclusion:**

Levothyroxine tablets combined with iodine-131 for thyroid cancer patients undergoing radical thyroidectomy effectively could improve the treatment efficiency, reduce the risk of cancer recurrence and metastasis after surgery, lower the serum Tg and TgAb levels of patients, and prolong the survival of patients, with a high safety profile. Further trials are, however, required prior to clinical promotion.

## 1. Introduction

Thyroid cancer is the most common thyroid malignancy tumor, including papillary carcinoma, follicular carcinoma, undifferentiated carcinoma, and medullary carcinoma. Papillary carcinoma is the most common in clinical practice and can be treated by surgery, which is usually supplemented by postoperative iodine-131 treatment and endocrine therapy to achieve better long-term treatment outcomes. Follicular carcinoma and medullary carcinoma are relatively rare, and undifferentiated carcinoma has the worst prognosis, which mainly relies on chemotherapy for treatment. Thyroid cancer is a thyroid malignancy derived from follicular epithelial cells with an incidence of about 1–5%, and the incidence in females is about 2.5 times that of men [1, 2]. It has been reported that postoperative thyroglobulin (Tg) and thyroglobulin antibody (TgAb) elevations in patients with thyroid cancer may increase the risk of recurrence. Serum Tg is a macromolecular glycoprotein secreted and produced by thyroid follicular epithelial cells, which is used to identify thyroid hypoplasia and complete defect and is also a tumor marker for differentiated thyroid cancer [3]. TgAb is an important thyroid tissue antibody and a diagnostic indicator for thyroid diseases. Its level is closely related to the severity of thyroid function injury. Levothyroxine tablets are mainly used for the treatment of hypothyroidism and hyperthyroidism and are also available for the inhibition (and replacement) therapy after thyroid cancer surgery and the inhibition test for the diagnosis of hyperthyroidism [4, 5]. Iodine-131 is a common drug with a significant effect in the treatment of differentiated thyroid cancer. It effectively improves the postoperative prognosis of patients and prevents metastasis and recurrence. It is currently the preferred postoperative treatment for differentiated thyroid cancer [6, 7]. This study recruited 70 patients with thyroid cancer receiving radical thyroidectomy in our hospital from July 2019 to April 2020 to explore the effectiveness of levothyroxine tablets combined with iodine-131 in thyroid cancer patients after radical thyroidectomy and its effect on serum Tg and TgAb levels to provide a reference for clinical practice.

## 2. Materials and Methods

### 2.1. General Information

A total of 70 thyroid cancer patients receiving radical thyroidectomy in our hospital from July 2015 to April 2016 were recruited and were assigned via different treatment methods (1 : 1) to either an observation group or a control group. In the observation group, there were 11 males and 24 females, aged 21–73 years, with a mean age of 48.27 ± 10.16 years. There were 21 cases of papillary carcinoma and 14 cases of follicular carcinoma in terms of cancer type, and there were 3 cases of grade I, 17 cases of grade II, 12 cases of grade III, and 3 cases of grade IV in terms of cancer grading. In the control group, there were 12 males and 23 females, aged 21–73 years, with an average age of 48.35 ± 10.20 years. There were 20 cases of papillary carcinoma and 15 cases of follicular carcinoma in terms of cancer type, and there were 2 cases of grade I, 15 cases of grade II, 14 cases of grade III, and 4 cases of grade IV in terms of cancer grading. There were no significant differences in gender, age, pathological type, and clinical-grade between the two groups of patients (*P* > 0.05), as given in Table 1. All patients and their families provided written informed consent, and the study was approved by the Ethical Review Committee of the Cangzhou Central Hospital, ERC04833.

### 2.2. Inclusion and Exclusion Criteria

Inclusion criteria were as follows: patients who were diagnosed with early thyroid cancer by CT and *X*-ray, with surgical indications, with solitary thyroid cancer that was confined to one lobe of the gland, with a lesion diameter of ≤1 cm, and with no lymph node and distal metastasis by a comprehensive assessment of imaging.

Exclusion criteria were as follows: patients with severe cardiopulmonary dysfunction, with autoimmune diseases, and with malignant tumors and intellectual or mental disorders.

### 2.3. Methods

All eligible patients received radical thyroidectomy for thyroid cancer. A longitudinal axillary incision of approximately 3-4 cm in length was made at the lateral border of the pectoralis major under the axilla, and the skin and subcutaneous fat were incised to expose the surface of the pectoralis major, and the pectoralis major was dissociated inward and upward along the pectoralis major fascia. The anterior cervical ligament muscle was freed medially, and the band was pulled up with a special retractor to fully expose the affected thyroid lobe, and the special retractor was fixed to the retractor frame to establish the surgical cavity.

The affected side, gland lobe, and isthmus were completely removed, and the specimens were sent for pathological examination. After the pathological diagnosis was confirmed, the central lymph nodes were removed, followed by the irrigation of the operative cavity, hemostasis, indwelling of the drainage tube, and suture of the wound.

The patients in the observation group received oral levothyroxine tablets (Shenzhen Zhonglian Pharmaceutical Co., Ltd., National Drug Approval H20010522) 7 days after surgery with a dose of 80–120 *μ*g/d, and the specific dose was based on the patient's thyroid stimulating hormone and thyroid hormone levels. The plasma thyrotropin level of patients with stage I was maintained at 0.1–0.3 mU/L, the plasma thyrotropin level of patients with stages II and III was maintained at 0.05–0.1 mU/L, and the plasma thyrotropin level of patients with stage IV was maintained at about 0.05 mU/L.

The patients in the control group received oral iodine-131 sodium chloride oral solution (Atomic Technology Co., Ltd., National Drug Approval H10960248), 1010Bq–660 × 1010Bq daily 30 days after surgery [8, 9]. After 6 months of continuous treatment, an ultrasound review was performed. Iodine-131 treatment was repeated once for those with residual or metastatic lesions of thyroid cancer. Patients in the control group also received a levothyroxine regimen similar to that in the observation group 7 days after the administration of iodine-131.

### 2.4. Observation Indicators

Treatment efficacy: markedly effective: lymph node metastasis disappeared completely, and no metastatic lymph nodes were developed; effective: lymph node mass was significantly reduced and softened, the area was reduced, and the mass activity was enhanced; ineffective: the tumor volume increased and the condition worsened. Total effective rate = (markedly effective + effective) number of cases/total number of cases × 100%. Cancer recurrence and metastasis: both groups of patients were followed up for 5 years after the operation. During the follow-up, the recurrence and metastasis of cancer cells in the two groups were recorded. After successful treatment, examinations of thyroglobulin (Tg), Tg antibody, *X*-ray, systemic iodine-131 imaging (I-WBS), and ultrasound were performed annually. If the results of I-WBS, *X*-ray, and ultrasound were negative, and the Tg level was less than 1.0 ug/L, and the patients were judged as no recurrence; otherwise, the conditions were judged as recurrence. Serum Tg and TgAb levels: the patients fasted for over 10 hours, and 5 mL of fasting venous blood was collected from the patients and centrifuged to obtain serum. The Roche Cobas e601 automatic electrochemiluminescence immunoassay was used to determine the levels of Tg and TgAb. The reagents used for the detection were all provided by Roche. The above steps were carried out strictly following the kit's instructions. Postoperative survival: the 1, 3, and 5-year survival of the two groups after the operation was recorded. Adverse reactions: adverse reactions including abnormal heart rate, fever, tremor, and rash were recorded.

### 2.5. Statistical Methods

SPSS 21.0 software was used for data analyses. Measurement data are expressed as mean ± SD and analyzed using the independent samples *t*-test. The count data are expressed as number of cases (rate) and analyzed using the chi-square test. Differences were considered statistically significant at *P* < 0.05.

## 3. Results

### 3.1. Comparison of Treatment Efficiency

The total effective rate of treatment in the control group was significantly higher than that in the observation group (*P* < 0.05), as given in Table 2.

### 3.2. Comparison of Cancer Recurrence and Metastasis

There was no significant difference in the recurrence and metastasis rate of cancer between the control group and the observation group 1 year after surgery (*P* > 0.05). The cancer recurrence and metastasis rates of the control group at 3 years and 5 years after surgery were significantly lower than those in the observation group (*P* < 0.05), as given in Table 3.

### 3.3. Comparison of Serum Tg and TgAb Levels before and after Treatment

Before treatment, there was no significant difference in serum Tg and TgAb levels between the two groups (*P* > 0.05). After treatment, the serum Tg and TgAb levels in the two groups decreased, and the control group had significantly lower results (*P* < 0.05), as given in Table 4.

### 3.4. Postoperative Survival

There was no significant difference in the 1-year and 3-year survival rates between the two groups (*P* > 0.05). The 5-year survival rate in the control group was significantly higher than that in the observation group (*P* < 0.05), as given in Table 5.

### 3.5. Comparison of Adverse Reactions

There was no significant difference in adverse reactions between the two groups (*P* > 0.05), as given in Table 6.

## 4. Discussion

At present, the etiology of thyroid cancer is poorly understood. Studies have shown that the pathogenesis of thyroid cancer is related to the lack of trace elements iodine, thyroid stimulating hormone stimulation, radiation stimulation, sex hormone stimulation, chemical, and genetic material [10, 11]. The clinical manifestations of thyroid cancer lack specificity. In the case of thyroid tumors, a hard texture, an uneven surface, and a nonmoving mass can be felt in the patient's thyroid. Patients with advanced cancer may be accompanied by symptoms such as dysphagia, dyspnea, hosing, Horner syndrome, shoulder and occipito-ear pain, and local or distal lymph node metastasis [12, 13]. Levothyroxine is mainly used for the treatment of hypothyroidism and hyperthyroidism in clinical practice and is available for suppressive therapy after thyroid cancer surgery [14, 15]. Iodine-131 is a relatively common drug in clinical practice with a significant effect in the treatment of differentiated thyroid cancer. It effectively improves the postoperative prognosis of patients and prevents the possibility of metastasis and recurrence. At present, surgery is the mainstay of treatment for thyroid cancer. Due to the slow progression of thyroid cancer and the low degree of malignancy, it has been reported that palliative treatment with thyroid hormones after tumor resection could achieve favorable results [16]. However, prior research has shown that most recurrence and metastasis occur within 5 years after surgery [17, 18], which was similar to the results of the present study. In the present study, the results showed that the total effective rate of the control group was significantly higher than that of the observation group; there was no significant difference in cancer recurrence and metastasis rate between the control group and the observation group one year after operation; the cancer recurrence and metastasis rates of the control group at 3 years and 5 years after surgery were significantly lower than those in the observation group; there was no significant difference in the survival rate of 3-year after surgery; the 5-year survival rate in the control group was significantly higher than that in the observation group. These results indicated that active postoperative treatment measures are essential for patients with thyroid cancer to reduce the risk of cancer recurrence and metastasis and improve their long-term survival. This also shows that levothyroxine tablets combined with iodine-131 therapy significantly improve the clinical treatment efficiency of patients and lower the risk of cancer recurrence and metastasis [17], thereby improving the long-term survival of patients [18, 19]. TgAb is an inhibitory autoimmune antibody produced by Tg. Zhang et al. demonstrated that thyroid cancer patients with elevated serum TgAb after radical thyroid cancer surgery had a high risk of recurrence during long-term follow-up, and the higher the serum TgAb level, the higher the risk of poor prognosis. Under normal circumstances, TgAb has little effect on thyroid tissue, but with Tg, it causes the interaction of Fc receptors, leading to the activation of natural killer cells and the destruction of thyroid cells. Therefore, the level of serum TgAb is associated with the level of Tg and the evaluation of the prognosis of thyroid cancer. Tg is a specific marker of thyroid tissue and is secreted by normal thyroid, benign thyroid diseases, and malignant tumors. Herein, the results showed that before treatment, there was no significant difference in the serum Tg and TgAb levels of the two groups of patients. After treatment, serum Tg and TgAb levels in both groups decreased, and the control group had lower results than the observation group, suggesting that levothyroxine tablets combined with iodine-131 could eliminate normal follicular cells and reduce the synthesis and level of Tg, thereby reducing antigen stimulation and TgAb level. This is consistent with the research results by Liang [20, 21]. In addition, there was no significant difference in the adverse reactions of the two groups of patients, which indicated that levothyroxine tablets combined with iodine-131 would not increase the adverse reactions of thyroid cancer patients.

In conclusion, levothyroxine tablets combined with iodine-131 for the treatment of thyroid cancer patients undergoing radical thyroidectomy could effectively improve the patients' treatment efficiency, reduce the risk of postoperative cancer recurrence and metastasis, control the patient's serum Tg and TgAb level, and prolong the survival of patients, with a high safety profile.

## Figures and Tables

**Table 1:** Comparison of general data (*n* (%)).

	Observation group (*n* = 35)	Control group (*n* = 35)	*t* or *x*^2^	*P*
Gender			0.065	0.799
Male	11	12		
Female	24	23		
Average age (years)	48.27 ± 10.16	48.35 ± 10.20	−0.033	0.974
The pathologic types			0.059	0.808
Papillary carcinoma	21	20		
Follicular carcinoma	14	15		
Clinical classification			0.215	0.643
Grade I	3	2		
Grade II	17	15		
Grade III	12	14		
Grade IV	3	4		

**Table 2:** Comparison of treatment efficiency (*n* (%)).

Group	Cases	Markedly effective	Effective	Ineffective	Total effective rate
Observation group	35	17	8	10	25 (71%)
Control group	35	19	14	2	33 (94%)
*x* ^2^	—	—	—	—	6.437
*P*	—	—	—	—	0.011

**Table 3:** Comparison of cancer recurrence and metastasis (*n* (%)).

Group	Cases	1 year after surgery	3 years after surgery	5 years after surgery
Observation group	35	3 (9%)	8 (26%)	11 (31%)
Control group	35	1 (3%)	2 (6%)	4 (11%)
*x* ^2^	—	2.832	10.857	7.925
*P*	—	0.092	0.001	0.005

**Table 4:** Comparison of serum Tg and TgAb levels before and after treatment (*x* ± *s*).

Group	Cases	Tg (ng/ml)		TgAb (IU/ml)	
		Preoperation	Postoperation	Preoperation	Postoperation
Observation group	35	157.34 ± 20.51	134.28 ± 15.21	1163.72 ± 230.45	1034.24 ± 168.73
Control group	35	158.21 ± 20.33	104.39 ± 18.91	1162.87 ± 229.78	841.15 ± 139.61
*t*	—	−0.178	7.287	0.015	5.216
*P*	—	0.859	＜0.001	0.988	＜0.001

**Table 5:** Postoperative survival (*n* (%)).

Group	Cases	1 year after surgery	3 years after surgery	5 years after surgery
Observation group	35	34 (97%)	30 (85%)	21 (60%)
Control group	35	35 (100%)	34 (97%)	31 (88%)
*x* ^2^	—	1.014	2.917	7.479
*P*	—	0.314	0.088	0.006

**Table 6:** Comparison of adverse reactions (*n* (%)).

	Observation group (*n* = 35)	Control group (*n* = 35)	*x* ^2^	*P*
Abnormal heart rate	2	3	—	—
Fever	1	0	—	—
Tremor	1	2	—	—
Rash	2	2	—	—
No adverse reaction	29	28	—	—
Total adverse reaction rate	6 (17%)	7 (20%)	0.094	0.759

## Data Availability

The datasets used during the present study are available from the corresponding author upon request.
